# Effectiveness of Transcranial Direct Current Stimulation and Monoclonal Antibodies Acting on the CGRP as a Combined Treatment for Migraine (TACTIC): Protocol for a Randomized, Double-Blind, Sham-Controlled Trial

**DOI:** 10.3389/fneur.2022.890364

**Published:** 2022-05-10

**Authors:** Raffaele Ornello, Chiara Rosignoli, Valeria Caponnetto, Francesca Pistoia, Michele Ferrara, Aurora D'Atri, Simona Sacco

**Affiliations:** Department of Biotechnological and Applied Clinical Sciences, University of L'Aquila, L'Aquila, Italy

**Keywords:** migraine treatment, transcranial direct current stimulation, electroencephalogram, monoclonal antibodies, calcitonin gene-related peptide, randomized controlled trials

## Abstract

**Background:**

Migraine is a recurrent headache disorder that has a still unclear pathophysiology, involving several circuits of both the central and peripheral nervous system. Monoclonal antibodies acting on the calcitonin gene-related (CGRP) pathway (CGRP-MAbs) are the first drugs specifically designed for migraine; those drugs act peripherally on the trigeminal ganglion without entering the blood-brain barrier. Conversely, neuromodulation techniques such as transcranial direct current stimulation (tDCS) act centrally by increasing or decreasing the neuronal firing rate of brain cortical areas. The aim of the study will be to evaluate whether tDCS, in addition to CGRP-MAbs, is an effective add-on treatment in reducing headache frequency, intensity and acute medication use in patients with migraine. To demonstrate the biological effects of tDCS, the electroencephalographic (EEG) power changes after tDCS will be assessed.

**Methods:**

We will include patients with migraine on treatment with CGRP-MAbs and reporting ≥8 monthly migraine days. During a prospective 28-day baseline period, patients will fill in a headache diary and questionnaires to evaluate migraine-related disability, anxiety and depressive symptoms, sleep quality, and health-related quality of life. Subjects will be randomly assigned in a 1:1 ratio to active or sham tDCS. The stimulation protocol will consist in five daily sessions, the cathodes will be applied bilaterally above the occipital areas, with the reference anode electrodes positioned above the primary motor areas. Before the first, and immediately after the last stimulation session, patients will perform a 10-min resting EEG recording. During a 28-day follow-up period following tDCS, patients will have to fill in a headache diary and questionnaires identical to those of the baseline period.

**Discussion:**

This trial will evaluate the efficacy of an add-on treatment acting on the brain in patients with migraine, who are already treated with peripherally acting drugs, showing how tDCS acts in restoring the dysfunctional brain networks typical of the migraine patient.

**Clinical Trial Registration:**

NCT05161871.

## Introduction

Migraine is a recurrent headache disorder representing the second cause of disability under 50 years of age and the first in young women (1). It is a central nervous system disorder whose mechanisms are poorly understood. Electrophysiology (EEG) studies have revealed abnormalities in cortical responsivity to external stimuli, in the different phases of the migraine cycle. During the days between attacks—i.e., the interictal period—, patients with migraine lack of habituation to external stimuli which normalizes in the hours that precede an attack (2). In the premonitory phase of migraine, changes in thalamo-cortical connectivity were observed; the presence of the so-called “thalamocortical dysrhythmia” is supported by MRI studies, before and during migraine attacks (3–5). Those studies showed altered connectivity of the cortex, thalamus, hypothalamus, brainstem and amygdala, which may be involved in the modulation of pain and sensory function (6).

Cortical spreading depression (CSD) is an event occurring in the brain which is supposed to play an important role in the genesis of migraine and to directly generate migraine aura (7). CSD consists in the diffusion of a wave of depolarization in the cerebral cortex that spreads slowly from the posterior areas of the brain; a “second phase” of neurophysiological and vascular changes ensues, characterized by a prolonged direct current potential shift that is lower in amplitude than the initial CSD wave, along with sustained vasoconstriction and reduced blood oxygenation (8). All those events lead to the activation of nociceptive centers, including a peripheral neural structure, the trigeminal ganglion (TG), which releases pain-inducing peptides and mostly calcitonin gene-related peptide (CGRP) (9, 10).

Several drug classes can be used for the prevention of migraine; they can be classified into antidepressants, antiepileptics, antihypertensives, onabotulinumtoxin A, beta-blockers, calcium agonists, and drugs that act on the calcitonin gene-related peptide (CGRP) pathway (11). Preventive drug treatment is not always viable due to potential contraindications; besides, patients may report adverse events or unsatisfactory benefit. Due to non-optimal adherence and poor tolerability to drugs, pharmacological preventive treatment can be replaced or integrated with non-pharmacological methods. Neuromodulation techniques, such as transcranial direct current stimulation (tDCS), are already used as a treatment for migraine and other chronic pain conditions (12–14), and it can be used in patients who prefer non-pharmacological management, or who cannot be adequately managed with drugs.

tDCS is a non-invasive and painless technique of brain modulation, consisting of delivering a weak current (1–2 mA) through two sponge electrodes fixed on the scalp and connected to a battery-driven stimulator; the aim of tDCS is to modulate spontaneous neuronal firing rate by the polarization of resting membrane potential (15). After-effects of the stimulation rely on the modulation of NMDA receptors and synaptic GABAergic activity (15). Anodal stimulation increases cortical excitability by depolarizing neurons in the stimulated area, while cathodal stimulation hyperpolarizes neurons with inhibitory effects (16).

Monoclonal antibodies acting on the CGRP pathway (CGRP-MAbs) are the first preventive drugs specifically designed for migraine; these drugs act by blocking the CGRP pathway, thereby inhibiting vasodilation and the transmission of pain (10). Those drugs demonstrated high efficacy in randomized controlled trials (17–19) and even more effectiveness in real-world studies (20–25). In real-life, the reduction in monthly migraine days due to CGRP-MAbs was up to 12.2 days at 6 months compared with baseline, while monthly days of acute medication consumption decreased up to 8; 50% response rates ranged from 10 to 76.5% (20–26). However, both randomized controlled trials and real-life studies showed that up to one half of patients in clinical practice do not attain a 50% reduction in monthly migraine days from baseline even with those specific treatments and need further improvements in their migraine prevention. Besides, many patients, even if reporting a significant response to those drugs, may have a high number of residual monthly headache days resulting in a substantial impact on daily activities (27). The number of residual monthly migraine days after treatment, although clinically relevant, is not reported by the available studies (28).

Due to their huge molecular dimensions, CGRP-MAbs inhibit CGRP release from the TG without crossing the blood-brain barrier (10); hence, they are not expected to interfere with the mechanisms of migraine occurring within the brain. On the contrary, tDCS acts on the central nervous system, by modulating the electrical activity of areas implied in pain modulation (29). Therefore, tDCS with CGRP-MAbs have different targets located at different levels in the nervous system; hence, we speculate that their combined administration can have a synergistic or additive effect.

## Objectives

The primary aim of the present study will be to assess whether tDCS as an add-on treatment to CGRP-MAbs is effective in reducing headache frequency, intensity, and acute medication use in patients with migraine. Secondarily, we will assess the effect of tDCS add-on on migraine-related disability, quality of life, sleep disturbance, and psychological symptoms. To demonstrate and quantify the biological effects of tDCS, we will assess the electroencephalographic (EEG) power changes after tDCS.

## Ethical Issues

The study was approved by the Ethics Committee for the districts of L'Aquila and Teramo with Protocol Number 272/21. All patients will sign an informed consent to participate in the study.

## Inclusion and Exclusion Criteria

Our trial will follow the guidelines issued by the International Headache Society for neuromodulation in headaches (30). The protocol follows the SPIRIT checklist (31, 32) (Supplementary Material 1). The inclusion criteria will be the following:

- male or female patients, aged between 40 and 70 years, referring to the Headache Center of the University of L'Aquila;
- a diagnosis of migraine with or without aura according to the International Classification of Headache Disorders, 3rd Edition (33);
- migraine must have been present for at least 12 months;
- treated with CGRP-MAbs (erenumab, fremanezumab or galcanezumab) for 90–180 days since the first subcutaneous administration (this time range was chosen to ensure a stable CGRP pathway inhibition);
- reporting ≥8 monthly migraine days in the last 30 days of observation despite treatment with CGRP-MAbs;
- able to discriminate between migraine and tension-type headaches;
- written informed consent to participate in the study.

Treatment with CGRP-MAbs will be prescribed according to Italian reimbursement criteria, i.e., in patients reporting ≥8 monthly migraine days with a Migraine Impact and Disability Assessment Scale score ≥11 and having failed at least three preventive medication classes among beta-blockers, tricyclic antidepressants, anticonvulsants, and onabotulinumtoxinA.

Patients with other concomitant primary headache types will be included if attacks are <1 day/month and <12 days/year.

Subjects with medication overuse headache and menstrually-related migraine will be not excluded from the study but will be included in exploratory subgroup analyses. According to the clinical practice of the recruiting center, patients with medication overuse will not undergo detoxication treatments.

The exclusion criteria will be the following:

- use of any concurrent migraine preventive medication other than CGRP-MAbs;
- secondary migraine-like headache;
- epilepsy or any other neurologic condition that may be worsened by transcranial electrical stimulation;
- metallic head implants, cardiac pacemaker or any other device that could malfunction or be displaced by electrical stimulation;
- pregnancy or lactation.

Acute migraine treatment will be allowed during the study. Migraine preventive treatments other than CGRP-MAbs must be withdrawn for at least 60 days before inclusion in the trial.

## Visit Schedule and Assessment

The study includes a 90- to 180-day retrospective screening period, a 28-day baseline period, a 5-day stimulation period, and a 28-day follow-up period. The planned inclusion period of the study will be 12 months. Assessment schedule is summarized in Table 1.

**Table 1:** Assessment schedule.

	**Screening**	**Baseline**	**Stimulation**	**Follow-up**
Informed consent	X			
Inclusion/exclusion criteria	X	X		
Clinical history	X	X		
Demographic data	X	X		
Headache diary	X*	X	X	X
mMIDAS		X		X
HIT-6		X		X
HADS		X		X
SF-36		X		X
PSQI		X		X
EEG		X	X	

CGRP-MAbs indicates monoclonal antibodies acting on the calcitonin gene-related peptide pathway; EEG, electroencephalogram; HADS, Hospital Anxiety and Assessment Scale; HIT-6, Headache Impact Test-6; mMIDAS, modified Migraine Impact and Disability Assessment Scale; PSQI, Pittsburgh Sleep Quality Index; SF-36, Short Form Health Survey. *Retrospective assessment based upon the Headache Center diary used in clinical practice.

At the beginning of the study, all subjects will be thoroughly informed about all aspects of the study, including the study treatment, visit schedule, required evaluations, diary compliance, and all regulatory requirements for informed consent. Subjects who sign an informed consent but fail to be assigned to the study treatment for any reason will be considered a screen failure. The reason for not being started on treatment will be recorded.

Subject demographic and baseline characteristic data will be collected on all subjects. This will include age, race, ethnicity, and relevant physiological and medical history. Prior headache characteristics and previous headache medication history, including information on the suitability for migraine prophylactics and prior migraine prophylactic treatment failure history, will be collected as part of screening and baseline characteristics.

## Randomization and Blinding

To control for placebo and nocebo effects, subjects will be randomly assigned in a 1:1 ratio to active or sham tDCS. Randomization will be performed by one of the investigators (AdA) unaware of personal data of study participants. A random allocation sequence will be generated in MATLAB environment; consecutive patients will then be allocated according to that sequence. The investigators who will administer the stimulation protocol (CR, RO), as well as the patient, will be blind as regards the type of stimulation applied (double blind). Finally, outcome assessment will be performed by an investigator (VC) blinded to the intervention performed.

## Study Procedures

### Baseline

Eligible subjects will undergo a 28-day baseline period to confirm their eligibility, by filling out a headache diary containing information about headache occurrence, its intensity on a 1–10 Numerical Rating Scale, its duration (in hours), associated symptoms (nausea, vomiting, photophobia, phonophobia), and consumption of drugs for the acute treatment. For each headache day, patients will have to rate their degree of headache-related disability as low-medium, or high (Supplementary Material 2).

At baseline, subjects will have to fill out questionnaires to assess migraine-related disability, quality of life, sleep disturbance and psychological aspects: the modified Migraine Disability Assessment (mMIDAS); the Headache Impact Test-6 (HIT-6); Short Form Health Survey (SF-36); Pittsburgh Sleep Quality Index (PSQI); Hospital Anxiety and Depression Scale (HADS).

### Stimulation Period

tDCS will be administered by trained personnel; one of the investigators (AdA) has years of experience in the tDCS field and will train two other investigators (CR, RO). The stimulation protocol will consist in five daily sessions, each lasting 20 min. The stimulation montage will provide a bilateral cathodal stimulation on occipital areas, with the reference anodal electrodes positioned on the M1 areas. The stimulation will be applied via 4 conductive-rubber square electrodes (5 × 5 cm) placed in sponges saturated with high conductivity gel and connected to a battery-operated stimulator system (BrainSTIM, EMS medical). In the active tDCS group, a direct current with maximal intensity of 1.5 mA with be provided for 20 mins (30 s ramp-in/ramp-out); those parameters are within the range of the available randomized controlled trials (34). In the sham group, the current will be turned off after 10 s (30 s ramp-in/ramp-out) at the beginning and at the end of the 20-min interval, in order to maintain the same tingling sensation that subjects refer during the gradual increase/decrease of the current intensity at the beginning/end of the ‘real' stimulation procedure. Patients will fill out the headache diary during the 5 days of tDCS.

### EEG Recording

Patients will perform a 10-min resting EEG recording (5 min eyes-open, 5-min eyes-closed), immediately before the first and immediately after the last tDCS session. EEG will be performed with a 64-channel apparel (BrainAmp, Brain Products GmbH) according to the 10–10 international system.

### Follow-Up

**Figure 1:**
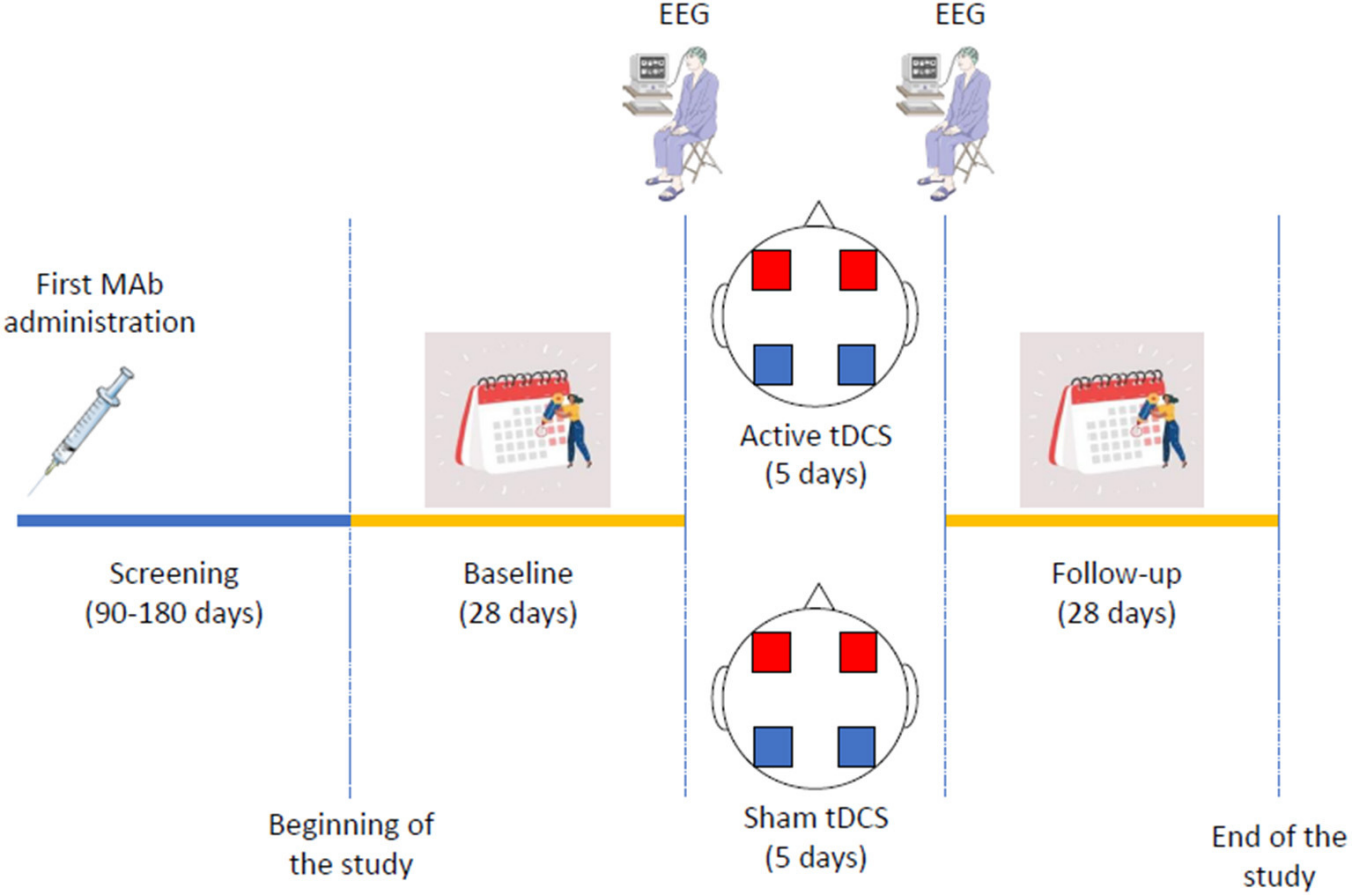
Schematic representation of trial procedures. Mab indicates monoclonal antibody; EEG, electroencephalogram; tDCS, transcranial direct current stimulation. In the transcranial direct current stimulation figure, blue squares indicate the cathodes, while red squares indicate the anodes.

Patients will undergo a 28-day follow-up assessment period starting from the day following the last tDCS session, filling out a diary identical to those of the baseline period. At the end of the follow-up period, patients will fill out the same questionnaires as during the baseline period. To verify blindness, patients will also be asked whether they received active or sham tDCS. The study procedures are summarized in Figure 1.

## Study Discontinuation

The study will be discontinued under the following circumstances:

- Subject decision;
- Pregnancy;
- Failed to meet the inclusion/exclusion criteria at any time during the study;
- Any situation in which study participation might result in a safety risk to the subject;
- Any change (initiation, withdrawal, or dosing change) in concurrent medication, including preventive and abortive treatment for migraine;
- New diagnosis of diseases that may be negatively affected by tDCS, such as epilepsy.

Study subjects will be consecutively recruited until the number of subjects completing the study reaches the number of 30 (15 treated with tDCS and 15 with sham stimulation). In case of screening failure or any of the conditions listed above and leading to study discontinuation, subjects will be replaced, provided that their inclusion falls within the 12-months inclusion period.

Patients discontinuing CGRP-MAbs due to non-response or lack of tolerance, as well as patients starting oral migraine preventive treatments as add-on, will be excluded from the study due to change in their medication. To ensure that treatment with CGRP-MAbs is stable and well-tolerated and to minimize the risk of including patients who will then withdraw treatment with CGRP-MAbs or change their medication, patient screening will be performed after 90–180 days from the first MAb administration. Patients lost to follow-up, unwilling to continue the trial, or developing a contraindication to continue the trial, will be excluded from efficacy analyses; their adverse events will be monitored and reported.

## Study Outcomes and Data Analysis

### Efficacy Outcomes

As we expect a short, 5-day course of tDCS to have a short-lasting effect on brain function, we will only assess short-term outcomes at 28 days after the end of tDCS.

The primary efficacy outcome will be the change in headache days from the 28-day baseline to the 28-day follow-up period.

The secondary outcomes will include the change in migraine days, headache hours, mean pain intensity (0–10 Visual Analog Scale), acute treatment consumption (doses), migraine-related disability (mMIDAS score) and impact (HIT-6 score), quality of life (SF-36 score), sleep quality (PSQI score), and anxious and depressive symptoms (HADS score) from the 28-day baseline to the 28-day follow-up period. The change in the number of days with low, medium, and high disability will also be assessed by using of a specifically designed headache diary (Supplementary Material 2).

The additional outcome will be the changes in spectral power and coherence in the delta (1–4 Hz), theta (5–7 Hz), alpha (8–12 Hz), and beta bands (13–30 Hz), both overall and over the occipital regions, at EEG recording between the two measurements (before vs. after tDCS). The EEG power changes will be correlated with the improvement in primary and secondary outcomes. The age limit of 40–70 years was chosen to limit the variability in EEG activity generated by the inclusion of too young or old subjects, which could act as a confounder in outcome assessment.

Each outcome will be assessed in the group of active and sham tDCS; additionally, between-group comparisons will be made.

A migraine day will be defined as a day with headache lasting at least 4 h if left untreated and accompanied by typical symptoms (nausea, vomiting, photo- and/or phonophobia) or preceded by aura. All days with headache not accompanied by any of those symptoms will be considered as non-migraine headache days. The total count of headache days will include both migraine and non-migraine headache days.

### Safety Outcomes

Safety assessment will include adverse event reporting. Adverse event monitoring will be performed during the tDCS stimulation sessions and during the 28-day follow-up period after tDCS. Adverse events will be detected and collected by investigators with a standard questionnaire (35) and open-ended questions. Monitoring for serious adverse events (SAEs) will be performed according to common clinical practice.

### Statistical Analysis

Continuous data will be summarized by mean, standard deviation (SD), median, first and third quartiles, minimum and maximum, Categorical data will be presented by absolute and relative frequencies (n and %). Bilateral 95% confidence limit will be presented as appropriate.

Comparison between groups (active/sham) for the variables under study (headache days, days of disabling headache, intensity of pain, consumption of acute treatments, headache-related disability, and scores on questionnaires) will be performed using parametric or non-parametric statistics, depending on the data distribution.

Primary analyses will be performed on primary and secondary outcomes. Exploratory subgroup analyses will be performed on patients with a history of menstrual migraine and on patients with chronic migraine with medication overuse.

To evaluate electrophysiological changes, the dependent variable will be the variations in EEG activity after vs. before tDCS. Specifically, we will compute the spectral power *via* Fast Fourier Transform (FFT) and the coherence in cortical activity among brain areas via magnitude-squared coherence (MSC) for the artifact-free epochs in each EEG frequency band. For each group of patients (tDCS vs. sham), the power change before vs. after tDCS will be compared for each electrode and each frequency band. Given the results of a previous study (36), particular attention will be given to power in the alpha band in occipital areas. The EEG index changes will be correlated with changes in migraine parameters (headache days, migraine days, pain intensity, acute medication consumption, questionnaires score) to directly link the modifications in brain physiology to the frequency and severity of migraine episodes. Source current density of cortical generators of relevant EEG indexes will be also assessed by low-resolution electromagnetic tomography (LORETA) (37), to confirm the cortical origin of the physiological changes induced by tDCS. Outcomes will be compared between the active and sham tDCS groups by chi-squared or t-test statistics as appropriate.

### Sample Size

The sample size calculation was performed using GPower, version 3.1. According to previous literature (36), a between-groups mean difference of 3 ± 2 migraine days per month was considered significant. The computation was made with the following parameters: confidence interval (two-sided): 95%; power: 80%; ratio of sample size: 1:1; mean change in group 1: −4 days; mean change in group 2: −1 day; standard deviation: 2. The minimum sample size suggested was of 9 patients per group. In consideration of possible dropouts, we set our population size to 30 patients, 15 per group.

## Discussion

CGRP-MAbs have significantly changed the landscape of migraine prevention. They are an effective and well tolerated class of drugs which can substantially improve the quality of life of patients with migraine. CGRP-MAbs were proved to be effective even in patients who had failures to other preventatives. The degree of benefit of CGRP-MAbs is highly variable; in up to 10% of patients they can lead to migraine freedom (100% responders) (17, 18, 38, 39), while in all the other patients there is a residual migraine impact despite the treatment. Some patients, even if meeting criteria to be considered as responders to CGRP-MAbs, continue to experience a significant burden of migraine. In fact, in real-life studies 14.4–57% of patients received add-on treatment (21, 22, 26). It is unclear which is the optimal treatment to be associated with CGRP-MAbs. We aim to evaluate if patients, with a significant migraine burden (>8 migraine days per month) despite the use of CGRP-MAbs, may achieve further benefit by adding a non-pharmacological (tDCS) treatment targeting central mechanisms involved in migraine. The rationale to choose tDCS is its non-pharmacological nature and the central mechanism of action which may be complementary to the peripheral mechanism of action of anti-CGRP-MAbs. We will randomize patients who are on treatment with CGRP-MAbs and who still experience a significant migraine burden (>8 migraine days per month) to tDCS or placebo.

So far, several studies have already evaluated tDCS for migraine prevention proving that it is a promising treatment to prevent migraine (36, 40–50). Available RCTs included a variable number of patients (from 15 to 135 patients) with highly heterogeneous patient populations, outcomes, time schedules, and tDCS montages (34). Most of the available RCTs performed either cathodal occipital stimulation with anterior reference (40, 43, 44, 46) or anodal frontal stimulation with supraorbital reference (36, 41, 42, 45). Those montages are both justified by neurophysiology, as studies on migraine showed a hyperresponsivity of the visual cortex, while frontal stimulation reduces the excitability of the thalamus, which is responsible for pain generation (29). Results of those RCTs were overall positive in the short term, while being more controversial 12 months after tDCS (36, 48). The available RCTs are limited by the underuse of neurophysiological tests, which would improve our understanding of the effect of tDCS and how to improve it (34).

With respect to the available RCTs, our study has several differences. Firstly, we will test tDCS as an add-on to a class of drugs specifically designed to prevent migraine. Besides, our montage will be bilateral with 4 electrodes (2 anodes and 2 cathodes), while the other trials all performed unilateral stimulation. The bilateral stimulation is justified by the supposed bilateral alterations of the migraine brain (4) and will likely optimize current flow through the brain. Moreover, our montage will merge cathodal occipital stimulation and anodal frontal stimulation, by positioning the cathode over both occipital regions and the anode over both frontal regions. Those procedures are intended to maximize neuromodulation of circuits involved in migraine and pain (29). Additionally, our study will follow as closely as possible the recently issued guidelines for trials of neuromodulation in patients with migraine (30).

We will also study the cortical effect of neuromodulation by electrophysiology (EEG). Previous studies have shown that EEG activity is different between subjects with and without migraine. In detail, migraineurs showed increased slow activity between attacks compared with non-migraineurs (51, 52) and the degree of EEG slowing on the occipital areas showed a correlation with the burden of migraine (53); the increase in slow activity is coupled with decreased power of the alpha frequency. Interestingly, a trial of anodal tDCS over the frontal motor areas showed that active treatment was associated with increased alpha power over the occipital regions (36), suggesting that tDCS can mitigate the neurophysiological abnormalities of the migraineurs' brain. Quantifying EEG activity in our trial will provide a neurophysiological correlate to clinical findings and will help explaining the effect of tDCS on neural structures. The use of high-density EEG will provide accurate information on the sites of tDCS action.

The present trial has a robust double-blind, randomized approach with blinded outcome assessment. The trial complies with the most recent guidelines for neuromodulation in migraine. Besides, the tDCS montage was designed specifically for migraine prevention by reflection on the most plausible neuroanatomical targets. However, the study also has limitations. The study is single-center; besides, its sample will be sufficient to calculate the primary outcome, while subgroup analyses will be only exploratory. We will correct for low numbers by assessing the normality of variable distributions and perform conservative, non-parametric tests for non-normally distributed variables. As an add-on to highly effective migraine preventatives, we cannot exclude that the clinical effect of tDCS in the present RCT will be negative. Nevertheless, we will include patients with a high burden of migraine (≥8 monthly migraine days) to correct for this effect. Besides, a previous trial showed that active tDCS is more effective than sham even on top of topiramate (47), suggesting that tDCS could be an effective add-on migraine preventative. Besides, we will assess not only the possible clinical efficacy of tDCS, but also its effect on brain circuitry; therefore, even results that are clinically neutral will be interesting to discuss with respect to the functional effects of tDCS. Our trial will contribute to assess the possible central, indirect effects of CGRP-MAbs by verifying whether the central circuits of migraine generation can be inhibited in addition to the action of those drugs.

## Conclusion

In conclusion, our trial will assess the efficacy of an add-on non-pharmacological treatment acting on the brain in patients with migraine who are already treated with peripherally acting CGRP-MAbs. The trial will also allow us to better understand the pathophysiology of migraine, and to evaluate how tDCS acts in restoring the dysfunctional brain networks typical of the migraine patient.

## Author Contributions

RO, AD'A, and SS conceived the study and wrote the protocol draft. All other authors provided critical revision to the draft. All authors read and approved the final protocol.

## Funding

This study will be partly funded by intramural DISCAB GRANT 2021 awarded by the Department of Biotechnological and Applied Clinical Sciences, University of L'Aquila.

## Conflict of Interest

RO declares personal fees from Novartis, Teva, and Eli Lilly, and non-financial support from Novartis, Allergan/AbbVie, and Teva. SS declares personal fees and non-financial support from Allergan, Abbott, Eli Lilly, Novartis, and Teva, personal fees from Medscape; and other from Bayer, Pfizer, Medtronic, Starmed, Bristol-Myers Squibb, and Daiichi Sankyo. The remaining authors declare that the research was conducted in the absence of any commercial or financial relationships that could be construed as a potential conflict of interest.

## Publisher's Note

All claims expressed in this article are solely those of the authors and do not necessarily represent those of their affiliated organizations, or those of the publisher, the editors and the reviewers. Any product that may be evaluated in this article, or claim that may be made by its manufacturer, is not guaranteed or endorsed by the publisher.

## Supplementary Material

Supplementary Material 1 SPIRIT checklist.

Supplementary Material 2 Headache diary.
