# Supplementary material for: Effectiveness of Transcranial Direct Current Stimulation and Monoclonal Antibodies Acting on the CGRP as a Combined Treatment for Migraine (TACTIC): Protocol for a Randomized, Double-Blind, Sham-Controlled Trial
Source: Front Neurol. 2022 May 10;13:890364. doi: 10.3389/fneur.2022.890364 (PMC9127506; doi:10.3389/fneur.2022.890364)
Supplement: Supplementary Material 1 — SPIRIT checklist. [file Data_Sheet_1.docx]

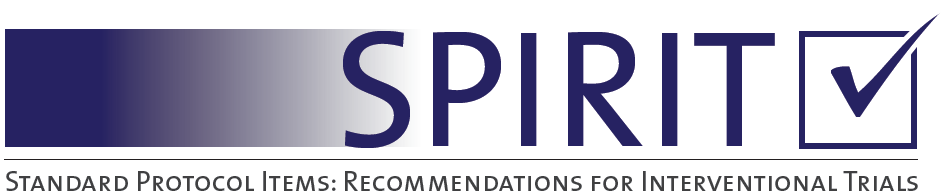


SPIRIT 2013 Checklist: Recommended items to address in a clinical trial protocol and related documents*

| Section/item | ItemNo | Description |
| --- | --- | --- |
| **Administrative information** | | |
| Title | 1 | Effectiveness of transcranial direct current stimulation and monoclonal antibodies acting on the CGRP as a combined treatment for migraine (TACTIC) |
| Trial registration | 2a | NCT05161871 (Clinicaltrials.gov) |
|  | 2b | All items from the World Health Organization Trial Registration Data Set |
| Protocol version | 3 | Version 00 (Original Protocol); date: 26-Aug-2021 |
| Funding | 4 | Department of Biotechnological and Applied Clinical Sciences, University of L’Aquila, Italy |
| Roles and responsibilities | 5a | Raffaele Ornello, Chiara Rosignoli, Valeria Caponnetto, Francesca Pistoia, Michele Ferrara, Aurora D’Atri, Simona Sacco, all from the Department of Biotechnological and Applied Clinical Sciences of the University of L’Aquila, Italy |
|  | 5b | Trial Sponsor: Simona Sacco, Department of Biotechnological and Applied Clinical Sciences, University of L’Aquila; Via Vetoio 1 – Coppito, 67100 L’Aquila Italy – [simona.sacco@univaq.it](mailto:simona.sacco@univaq.it) |
|  | 5c | The sponsor will have a primary role in: study design; collection, management, analysis, and interpretation of data; writing of the report; and the decision to submit the report for publication. The funder will support the supply of study materials. |
|  | 5d | SS and MF will coordinate the study; AdA, RO, and CR will oversee data collection; VC will adjudicate outcome and perform data analysis; FP will perform data monitoring. |
| Introduction |  |  |
| Background and rationale | 6a | Migraine pathophysiology involves both the central and peripheral nervous system (doi: 10.1152/physrev.00034.2015). Monoclonal antibodies acting on the calcitonin gene-related (CGRP) pathway (CGRP-MAbs) are the first drugs specifically designed for migraine; those drugs act peripherally on the trigeminal ganglion without entering the blood-brain barrier (doi: 10.1038/s41582-018-0003-1). Conversely, neuromodulation techniques such as transcranial direct current stimulation (tDCS) act centrally by increasing or decreasing the neuronal firing rate of brain cortical areas (doi: 10.3389/fpsyt.2012.00110). Therefore, we hypothesize that tDCS is an effective add-on treatment to CGRP-MAbs in reducing headache frequency, intensity and acute medication use in patients with migraine. |
|  | 6b | We will perform a comparison between active and sham tDCS in patients already on treatment with CGRP-MAbs; the comparison between an active and a sham procedure will ensure accuracy of measurements. We will also perform an objective assessment of brain function before and after neuromodulation through electroencephalogram (EEG). |
| Objectives | 7 | Primary aim: to assess whether tDCS as an add-on treatment to CGRP-MAbs is effective in reducing headache frequency, intensity, and acute medication use in patients with migraine. Secondary aim: to assess the effect of tDCS add-on on migraine-related disability, quality of life, sleep disturbance, and psychological symptoms. Additional aim: to assess the EEG power changes after tDCS. |
| Trial design | 8 | This is a parallel group, randomized, double-blind trial with 1:1 allocation ratio; analyses will be exploratory. |
| Methods: Participants, interventions, and outcomes | | |
| Study setting | 9 | The trial will be single-center and will be performed in an academc hospital. |
| Eligibility criteria | 10 | The inclusion criteria will be the following:  - male or female patients, aged between 40 and 70 years, referring to the Headache Center of the University of L’Aquila;  -a diagnosis of migraine with or without aura according to the International Classification of Headache Disorders, 3rd Edition (2018);  - migraine must have been present for at least 12 months;  - treated with CGRP-MAbs (erenumab, fremanezumab or galcanezumab) for 90-180 days since the first subcutaneous administration (this time range was chosen to ensure a stable CGRP pathway inhibition);  - reporting ≥8 monthly migraine days in the last 30 days of observation despite treatment with CGRP-MAbs;  - able to discriminate between migraine and tension-type headaches;  - written informed consent to participate in the study.  Patients with other concomitant primary headache types will be included if attacks are <1 day/month and <12 days/year.  Subjects with medication overuse headache and menstrually-related migraine will be not excluded from the study but will be included in exploratory subgroup analyses. According to the clinical practice of the recruiting center, patients with medication overuse will not undergo detoxication treatments.  The exclusion criteria will be the following:  - use of any concurrent migraine preventive medication other than CGRP-MAbs;  - secondary migraine-like headache;  - epilepsy or any other neurologic condition that may be worsened by transcranial electrical stimulation;  - metallic head implants, cardiac pacemaker or any other device that could malfunction or be displaced by electrical stimulation;  - pregnancy or lactation.  Acute migraine treatment will be allowed during the study. Migraine preventive treatments other than CGRP-MAbs must be withdrawn for at least 60 days before inclusion in the trial. |
| Interventions | 11a | Eligible subjects will undergo a 28-day baseline period to confirm their eligibility, by filling out a headache diary containing information about headache occurrence, its intensity on a 1-10 Numerical Rating Scale, its duration (in hours), associated symptoms (nausea, vomiting, photophobia, phonophobia), and consumption of drugs for the acute treatment. For each headache day, patients will have to rate their degree of headache-related disability as low-medium, or high (Supplementary File 2).  At baseline, subjects will have to fill out questionnaires to assess migraine-related disability, quality of life, sleep disturbance and psychological aspects: the modified Migraine Disability Assessment (mMIDAS); the Headache Impact Test-6 (HIT-6); Short Form Health Survey (SF-36); Pittsburgh Sleep Quality Index (PSQI); Hospital Anxiety and Depression Scale (HADS).  7.2 Stimulation period  tDCS will be administered by trained personnel; one of the investigators (AdA) has years of experience in the tDCS field and will train two other investigators (CR, RO). The stimulation protocol will consist in five daily sessions, each lasting 20 min. The stimulation montage will provide a bilateral cathodal stimulation on occipital areas, with the reference anodal electrodes positioned on the M1 areas. The stimulation will be applied via 4 conductive-rubber square electrodes (5x5 cm) placed in sponges saturated with high conductivity gel and connected to a battery-operated stimulator system (BrainSTIM, EMS medical). In the active tDCS group, a direct current with maximal intensity of 1.5 mA with be provided for 20 minutes (30 sec ramp-in/ramp-out). In the sham group, the current will be turned off after 10 sec (30 sec ramp-in/ramp-out) at the beginning and at the end of the 20-min interval, in order to maintain the same tingling sensation that subjects refer during the gradual increase/decrease of the current intensity at the beginning/end of the ‘real’ stimulation procedure. Patients will fill out the headache diary during the five days of tDCS.  Patients will perform a 10-min resting EEG recording (5 min eyes-open, 5-min eyes-closed), immediately before the first and immediately after the last tDCS session. EEG will be performed with a 64-channel apparel (BrainAmp, Brain Products GmbH) according to the 10-10 international system.  Patients will undergo a 28-day follow-up assessment period starting from the day following the last tDCS session, filling out a diary identical to those of the baseline period. At the end of the follow-up period, patients will fill out the same questionnaires as during the baseline period. The study procedures are summarized in Figure 1. |
|  | 11b | The study will be discontinued under the following circumstances:  - Subject decision;  - Pregnancy;  - Failed to meet the inclusion/exclusion criteria at any time during the study;  - Any situation in which study participation might result in a safety risk to the subject;  - Any change (initiation, withdrawal, or dosing change) in concurrent medication, including preventive and abortive treatment for migraine;  - New diagnosis of diseases that may be negatively affected by tDCS, such as epilepsy.  Study subjects will be consecutively recruited until the number of subjects completing the study reaches the number of 30 (15 treated with tDCS and 15 with sham stimulation). In case of screening failure or any of the conditions listed above and leading to study discontinuation, subjects will be replaced, provided that their inclusion falls within the 12-months inclusion period.  Patients discontinuing CGRP-MAbs due to non-response or lack of tolerance, as well as patients starting oral migraine preventive treatments as add-on, will be excluded from the study due to change in their medication. To ensure that treatment with CGRP-MAbs is stable and well-tolerated and to minimize the risk of including patients who will then withdraw treatment with CGRP-MAbs or change their medication, patient screening will be performed after 90-180 days from the first MAb administration. Patients lost to follow-up, unwilling to continue the trial, or developing a contraindication to continue the trial, will be excluded from efficacy analyses; their adverse events will be monitored and reported. |
|  | 11c | Adherence to the study interventions will be ensured as the interventions will be performed at the study center. |
|  | 11d | Patients will be allowed to continue their usual medication, at the condition of not changing them throughout the study. |
| Outcomes | 12 | Primary efficacy outcome: change in headache days from the 28-day baseline to the 28-day follow-up period.  Secondary efficacy outcomes: change in migraine days, headache hours, mean pain intensity (0-10 Visual Analog Scale), acute treatment consumption (doses), migraine-related disability (mMIDAS score) and impact (HIT-6 score), quality of life (SF-36 score), sleep quality (PSQI score), and anxious and depressive symptoms (HADS score) from the 28-day baseline to the 28-day follow-up period. The change in the number of days with low, medium, and high disability will also be assessed by using of a specifically designed headache diary.  Additional efficacy outcome: changes in spectral power and coherence in the delta (1-4 Hz), theta (5-7 Hz), alpha (8-12 Hz), and beta bands (13-30 Hz), both overall and over the occipital regions, at EEG recording between the two measurements (before vs after tDCS). The EEG power changes will be correlated with the improvement in primary and secondary outcomes.  Each outcome will be assessed in the group of active and sham tDCS; additionally, between-group comparisons will be made.  Safety outcome: adverse events; serious adverse events (SAEs). |
| Participant timeline | 13 | The study timeline is summarized in the Figure below:  The study timeline is reported in the Figure below: 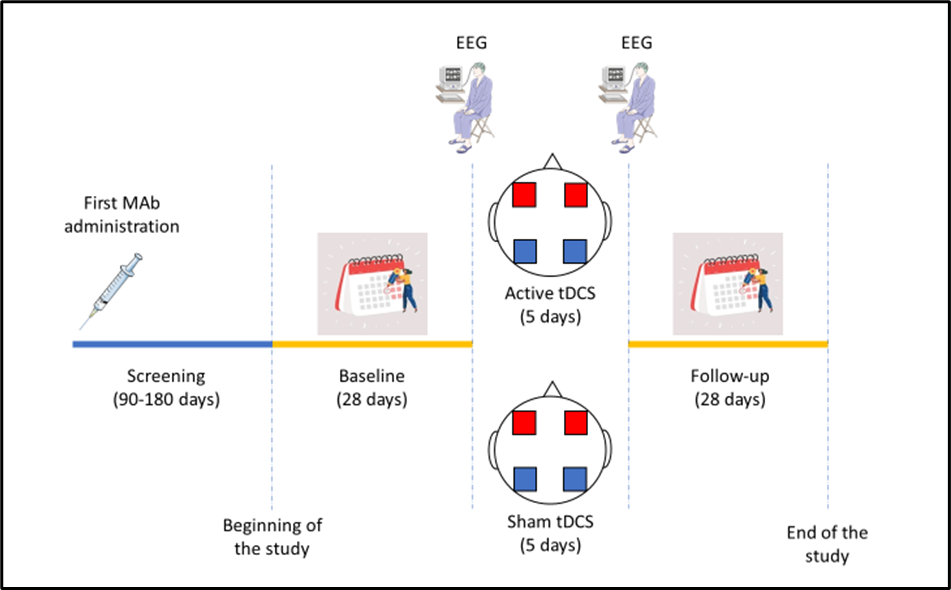 |
| Sample size | 14 | The sample size calculation was performed using GPower, version 3.1. According to previous literature (doi:10.1016/j.clinph.2020.10.014), a between-groups mean difference of 3±2 migraine days per month was considered significant. The computation was made with the following parameters: confidence interval (two-sided): 95%; power: 80%; ratio of sample size: 1:1; mean change in group 1: -4 days; mean change in group 2: -1 day; standard deviation: 2. The minimum sample size suggested was of 9 patients per group. In consideration of possible dropouts, we set our population size to 30 patients, 15 per group. |
| Recruitment | 15 | The sample size will be achieved through consecutive recruitment of patients in the Headache Center of the University of L’Aquila. This is a comprehensive Headache Center and a prescription center of CGRP-MAbs. |
| **Methods: Assignment of interventions (for controlled trials)** | | |
| Allocation: |  |  |
| Sequence generation | 16a | The allocation sequence will be generated randomly with a computer software before randomization. No stratification will be performed. |
| Allocation concealment mechanism | 16b | Thirty tDCS stimulation protocols will be generated, one for each patient, sequentially numbered, and stored in a computer. The investigators performing active or sham tDCS will be blind to the interventions as well as the patients. |
| Implementation | 16c | AdA will generate the allocation sequence; SS will enrol participants; CR and RO will assign participants to interventions according to the numbered random sequence. |
| Blinding (masking) | 17a | Trial participants, care providers, and outcome assessors will be blinded to the intervention |
|  | 17b | Unblinding will be allowed only in case of urgent medical necessities or if requested by the patients because of medical needs. Unblinded patients will be excluded from the study. |
| **Methods: Data collection, management, and analysis** | | |
| Data collection methods | 18a | Data will be collected by personnel with experience in randomized clinical trials. Data collection will include demographic data, headache diary data, and questionnaires referring to migraine-related disability, sleep quality, and health-related quality of life. Data will be collected on paper and reported electronically through Research Electronic Data Capture (REDCap). EEG data will be collected by computers and elaborated by computer software (MATLAB). |
|  | 18b | All data, including those pf patients discontinuing the study, will be collected throughout the study period. Participant retention and study completion will be encouraged throughout the study period by means of periodic reminders. |
| Data management | 19 | Data entry will be performed by the investigators performing tDCS (RO and CR) and checked by the outcome assessor (VC). Data cleaning will be performed with queries. |
| Statistical methods | 20a | Continuous data will be summarized by mean, standard deviation (SD), median, first and third quartiles, minimum and maximum, Categorical data will be presented by absolute and relative frequencies (n and %). Bilateral 95% confidence limit will be presented as appropriate.  Comparison between groups (active/sham) for the variables under study (headache days, days of disabling headache, intensity of pain, consumption of acute treatments, headache-related disability, and scores on questionnaires) will be performed using parametric or non-parametric statistics, depending on the data distribution.  Primary analyses will be performed on primary and secondary outcomes. Exploratory subgroup analyses will be performed on patients with a history of menstrual migraine and on patients with chronic migraine with medication overuse.  To evaluate electrophysiological changes, the dependent variable will be the variations in EEG activity after vs before tDCS. Specifically, we will compute the spectral power via Fast Fourier Transform (FFT) and the coherence in cortical activity among brain areas via magnitude-squared coherence (MSC) for the artifact-free epochs in each EEG frequency band. For each group of patients (tDCS vs sham), the power change before vs after tDCS will be compared for each electrode and each frequency band. The EEG index changes will be correlated with changes in migraine parameters (headache days, migraine days, pain intensity, acute medication consumption, questionnaires score) to directly link the modifications in brain physiology to the frequency and severity of migraine episodes. Source current density of cortical generators of relevant EEG indexes will be also assessed by low-resolution electromagnetic tomography (LORETA) to confirm the cortical origin of the physiological changes induced by tDCS. Outcomes will be compared between the active and sham tDCS groups by chi-squared or t-test statistics as appropriate. |
|  | 20b | No subgroup analyses are planned. |
|  | 20c | Data will be analyzed for the intention-to-treat population. Missing data will be imputed with a “last-observation-carried-forward” approach. |
| **Methods: Monitoring** | | |
| Data monitoring | 21a | A Data Monitoring Committee is not needed due to the single-center nature of the study. A single investigator (FP) will monitor the study data. |
|  | 21b | No interim analysis is planned. |
| Harms | 22 | Adverse events will be collected at each study visit and as soon as they come to the investigators’ attention. Adverse events will be reported to the local Health Autorities and be managed according to local clinical practice. |
| Auditing | 23 | No auditing is planned. |
| Ethics and dissemination | | |
| Research ethics approval | 24 | The study was approved by the local Ethics Committee. |
| Protocol amendments | 25 | All important protocol modifications will be notified to the local Ethics Committee. |
| Consent or assent | 26a | Written informed consent will be obtained by the recruiting study staff (RO, SS, CR). |
|  | 26b | No additional consent is planned as there will be no ancillary study. |
| Confidentiality | 27 | Personal information will be collected and stored securely in a closed room and in a password-protected system. Access to patient information will only be allowed to the study staff. |
| Declaration of interests | 28 | No conflicts of interest are related to the present study. |
| Access to data | 29 | All the study staff will have access to the final trial dataset after data analysis. The study datasets will be available from the study staff members upon reasonable request after publication of the study results. |
| Ancillary and post-trial care | 30 | No serious adverse event is anticipated. Potential harms to patients will be covered by the public insurance system of the study center. Post-trial care will follow common clinical practice. |
| Dissemination policy | 31a | Trial results will be communicated to healthcare professionals via papers and conference communications. Participants and the public will receive communication of the trial results via plain language summaries and social media posts after peer-reviewed publication of the trial results. |
|  | 31b | The study staff is eligible to authorship; any other researcher who will provide a significant intellectual contribution to the study development or paper writing will be eligible as Author after authorization by the study staff. No use of professional writers is planned. |
|  | 31c | Plans, if any, for granting public access to the full protocol, participant-level dataset, and statistical code |
| Appendices |  |  |
| Informed consent materials | 32 | Consent form is available upon reasonable request from the study staff. |
| Biological specimens | 33 | Not applicable. |

*It is strongly recommended that this checklist be read in conjunction with the SPIRIT 2013 Explanation & Elaboration for important clarification on the items. Amendments to the protocol should be tracked and dated. The SPIRIT checklist is copyrighted by the SPIRIT Group under the Creative Commons “[Attribution-NonCommercial-NoDerivs 3.0 Unported](http://www.creativecommons.org/licenses/by-nc-nd/3.0/)” license.
