# Supplementary material for: Effectiveness of Transcranial Direct Current Stimulation and Monoclonal Antibodies Acting on the CGRP as a Combined Treatment for Migraine (TACTIC): Protocol for a Randomized, Double-Blind, Sham-Controlled Trial
Source: Front Neurol. 2022 May 10;13:890364. doi: 10.3389/fneur.2022.890364 (PMC9127506; doi:10.3389/fneur.2022.890364)
Supplement: Supplementary Material 2 — Headache diary. [file Data_Sheet_2.docx]

**Supplementary File 2**. Headache diary.

Patient code: |__|__|__|

When a headache occurs, please write indicate:

- If you have ‘non-migraine attack’ (**Headache**) or ‘migraine attack’ (**Migraine**)

**TO CLARIFY, find below the characteristics of migraine attacks**:

1. **Headache associated at least with one of the following symptoms**: nausea, vomiting, phonophobia, photophobia.
2. **Pain has at least one of the following characteristics**: moderate or severe pain intensity, aggravation by routine physical activity, unilateral location, pulsating quality.

**When these two features are not present, you will select ‘Headache’ as attack type.**

- Select ‘**Aura**’ if you also experienced an **aura**
- Indicate **pain intensity** from 1 to 10, where:

|  |  |  |  |  |  |  |  |  |  |
| --- | --- | --- | --- | --- | --- | --- | --- | --- | --- |
| **1** | 2 | 3 | 4 | 5 | 6 | 7 | 8 | 9 | **10** |
| **Very minor pain** | |  |  |  |  |  |  | **Bigger pain**  **possible** | |

- Indicate **pain duration** in hours
- Indicate the trade name of the analgesic you took (if taken) and its effectiveness: ‘**Y**’ if you had **complete pain** **relief**; ‘**P**’ if you had **partial pain relief**; ‘**No**’ if you did **not have pain relief** at all
- Add the **headache impact on daily-life activities**, referring to work, home, and recreational activities scheduled for the day in which headache occurs. Select the following symbols:

| **Symbol** | **Description** | |
| --- | --- | --- |
| ⏺ | 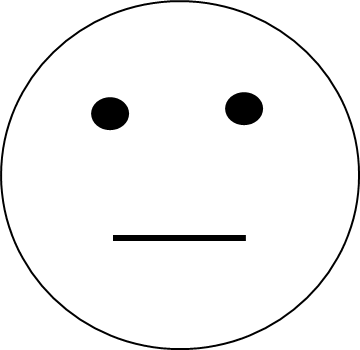 | **Low disability:** despite headache, you are able to perform your scheduled activities; your general efficiency is not affected |
| ⏺⏺ | 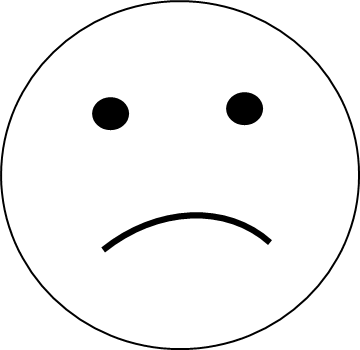 | **Medium disability**: because of headache, you are able to perform only some scheduled activities and you have to give up some activities; your general efficiency is reduced |
| ⏺⏺⏺ | 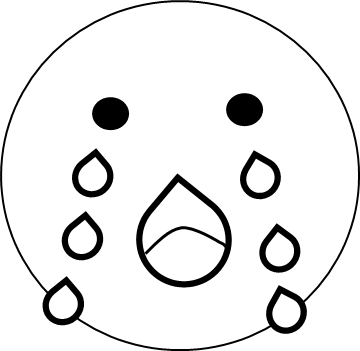 | **High disability**: because of headache, you are not able to perform your scheduled activities and you have to give up everything; your general efficiency is highly affected |

| Day | Attack features | Intensity  1 to 10 | Lasting hours | Analgesics trade name (if taken) | Effectiveness | Disability |
| --- | --- | --- | --- | --- | --- | --- |
| 1 | [ ] Headache  [ ] Migraine  [ ] Aura |  |  | _______________  _______________  _______________ | [ ] Y [ ] P [ ] No  [ ] Y [ ] P [ ] No  [ ] Y [ ] P [ ] No | [ ] ⏺  [ ] ⏺⏺  [ ] ⏺⏺⏺ |
| 2 | [ ] Headache  [ ] Migraine  [ ] Aura |  |  | _______________  _______________  _______________ | [ ] Y [ ] P [ ] No  [ ] Y [ ] P [ ] No  [ ] Y [ ] P [ ] No | [ ] ⏺  [ ] ⏺⏺  [ ] ⏺⏺⏺ |
| 3 | [ ] Headache  [ ] Migraine  [ ] Aura |  |  | _______________  _______________  _______________ | [ ] Y [ ] P [ ] No  [ ] Y [ ] P [ ] No  [ ] Y [ ] P [ ] No | [ ] ⏺  [ ] ⏺⏺  [ ] ⏺⏺⏺ |
| 4 | [ ] Headache  [ ] Migraine  [ ] Aura |  |  | _______________  _______________  _______________ | [ ] Y [ ] P [ ] No  [ ] Y [ ] P [ ] No  [ ] Y [ ] P [ ] No | [ ] ⏺  [ ] ⏺⏺  [ ] ⏺⏺⏺ |
| 5 | [ ] Headache  [ ] Migraine  [ ] Aura |  |  | _______________  _______________  _______________ | [ ] Y [ ] P [ ] No  [ ] Y [ ] P [ ] No  [ ] Y [ ] P [ ] No | [ ] ⏺  [ ] ⏺⏺  [ ] ⏺⏺⏺ |
| 6 | [ ] Headache  [ ] Migraine  [ ] Aura |  |  | _______________  _______________  _______________ | [ ] Y [ ] P [ ] No  [ ] Y [ ] P [ ] No  [ ] Y [ ] P [ ] No | [ ] ⏺  [ ] ⏺⏺  [ ] ⏺⏺⏺ |
| 7 | [ ] Headache  [ ] Migraine  [ ] Aura |  |  | _______________  _______________  _______________ | [ ] Y [ ] P [ ] No  [ ] Y [ ] P [ ] No  [ ] Y [ ] P [ ] No | [ ] ⏺  [ ] ⏺⏺  [ ] ⏺⏺⏺ |
| 8 | [ ] Headache  [ ] Migraine  [ ] Aura |  |  | _______________  _______________  _______________ | [ ] Y [ ] P [ ] No  [ ] Y [ ] P [ ] No  [ ] Y [ ] P [ ] No | [ ] ⏺  [ ] ⏺⏺  [ ] ⏺⏺⏺ |
| 9 | [ ] Headache  [ ] Migraine  [ ] Aura |  |  | _______________  _______________  _______________ | [ ] Y [ ] P [ ] No  [ ] Y [ ] P [ ] No  [ ] Y [ ] P [ ] No | [ ] ⏺  [ ] ⏺⏺  [ ] ⏺⏺⏺ |
| 10 | [ ] Headache  [ ] Migraine  [ ] Aura |  |  | _______________  _______________  _______________ | [ ] Y [ ] P [ ] No  [ ] Y [ ] P [ ] No  [ ] Y [ ] P [ ] No | [ ] ⏺  [ ] ⏺⏺  [ ] ⏺⏺⏺ |
| 11 | [ ] Headache  [ ] Migraine  [ ] Aura |  |  | _______________  _______________  _______________ | [ ] Y [ ] P [ ] No  [ ] Y [ ] P [ ] No  [ ] Y [ ] P [ ] No | [ ] ⏺  [ ] ⏺⏺  [ ] ⏺⏺⏺ |
| 12 | [ ] Headache  [ ] Migraine  [ ] Aura |  |  | _______________  _______________  _______________ | [ ] Y [ ] P [ ] No  [ ] Y [ ] P [ ] No  [ ] Y [ ] P [ ] No | [ ] ⏺  [ ] ⏺⏺  [ ] ⏺⏺⏺ |
| 13 | [ ] Headache  [ ] Migraine  [ ] Aura |  |  | _______________  _______________  _______________ | [ ] Y [ ] P [ ] No  [ ] Y [ ] P [ ] No  [ ] Y [ ] P [ ] No | [ ] ⏺  [ ] ⏺⏺  [ ] ⏺⏺⏺ |
| 14 | [ ] Headache  [ ] Migraine  [ ] Aura |  |  | _______________  _______________  _______________ | [ ] Y [ ] P [ ] No  [ ] Y [ ] P [ ] No  [ ] Y [ ] P [ ] No | [ ] ⏺  [ ] ⏺⏺  [ ] ⏺⏺⏺ |
| 15 | [ ] Headache  [ ] Migraine  [ ] Aura |  |  | _______________  _______________  _______________ | [ ] Y [ ] P [ ] No  [ ] Y [ ] P [ ] No  [ ] Y [ ] P [ ] No | [ ] ⏺  [ ] ⏺⏺  [ ] ⏺⏺⏺ |
| 16 | [ ] Headache  [ ] Migraine  [ ] Aura |  |  | _______________  _______________  _______________ | [ ] Y [ ] P [ ] No  [ ] Y [ ] P [ ] No  [ ] Y [ ] P [ ] No | [ ] ⏺  [ ] ⏺⏺  [ ] ⏺⏺⏺ |

| Day | Attack features | Intensity  1 to 10 | Lasting hours | Analgesics trade name (if took) | Effectiveness | Disability |
| --- | --- | --- | --- | --- | --- | --- |
| 17 | [ ] Headache  [ ] Migraine  [ ] Aura |  |  | _______________  _______________  _______________ | [ ] Y [ ] P [ ] No  [ ] Y [ ] P [ ] No  [ ] Y [ ] P [ ] No | [ ] ⏺  [ ] ⏺⏺  [ ] ⏺⏺⏺ |
| 18 | [ ] Headache  [ ] Migraine  [ ] Aura |  |  | _______________  _______________  _______________ | [ ] Y [ ] P [ ] No  [ ] Y [ ] P [ ] No  [ ] Y [ ] P [ ] No | [ ] ⏺  [ ] ⏺⏺  [ ] ⏺⏺⏺ |
| 19 | [ ] Headache  [ ] Migraine  [ ] Aura |  |  | _______________  _______________  _______________ | [ ] Y [ ] P [ ] No  [ ] Y [ ] P [ ] No  [ ] Y [ ] P [ ] No | [ ] ⏺  [ ] ⏺⏺  [ ] ⏺⏺⏺ |
| 20 | [ ] Headache  [ ] Migraine  [ ] Aura |  |  | _______________  _______________  _______________ | [ ] Y [ ] P [ ] No  [ ] Y [ ] P [ ] No  [ ] Y [ ] P [ ] No | [ ] ⏺  [ ] ⏺⏺  [ ] ⏺⏺⏺ |
| 21 | [ ] Headache  [ ] Migraine  [ ] Aura |  |  | _______________  _______________  _______________ | [ ] Y [ ] P [ ] No  [ ] Y [ ] P [ ] No  [ ] Y [ ] P [ ] No | [ ] ⏺  [ ] ⏺⏺  [ ] ⏺⏺⏺ |
| 22 | [ ] Headache  [ ] Migraine  [ ] Aura |  |  | _______________  _______________  _______________ | [ ] Y [ ] P [ ] No  [ ] Y [ ] P [ ] No  [ ] Y [ ] P [ ] No | [ ] ⏺  [ ] ⏺⏺  [ ] ⏺⏺⏺ |
| 23 | [ ] Headache  [ ] Migraine  [ ] Aura |  |  | _______________  _______________  _______________ | [ ] Y [ ] P [ ] No  [ ] Y [ ] P [ ] No  [ ] Y [ ] P [ ] No | [ ] ⏺  [ ] ⏺⏺  [ ] ⏺⏺⏺ |
| 24 | [ ] Headache  [ ] Migraine  [ ] Aura |  |  | _______________  _______________  _______________ | [ ] Y [ ] P [ ] No  [ ] Y [ ] P [ ] No  [ ] Y [ ] P [ ] No | [ ] ⏺  [ ] ⏺⏺  [ ] ⏺⏺⏺ |
| 25 | [ ] Headache  [ ] Migraine  [ ] Aura |  |  | _______________  _______________  _______________ | [ ] Y [ ] P [ ] No  [ ] Y [ ] P [ ] No  [ ] Y [ ] P [ ] No | [ ] ⏺  [ ] ⏺⏺  [ ] ⏺⏺⏺ |
| 26 | [ ] Headache  [ ] Migraine  [ ] Aura |  |  | _______________  _______________  _______________ | [ ] Y [ ] P [ ] No  [ ] Y [ ] P [ ] No  [ ] Y [ ] P [ ] No | [ ] ⏺  [ ] ⏺⏺  [ ] ⏺⏺⏺ |
| 27 | [ ] Headache  [ ] Migraine  [ ] Aura |  |  | _______________  _______________  _______________ | [ ] Y [ ] P [ ] No  [ ] Y [ ] P [ ] No  [ ] Y [ ] P [ ] No | [ ] ⏺  [ ] ⏺⏺  [ ] ⏺⏺⏺ |
| 28 | [ ] Headache  [ ] Migraine  [ ] Aura |  |  | _______________  _______________  _______________ | [ ] Y [ ] P [ ] No  [ ] Y [ ] P [ ] No  [ ] Y [ ] P [ ] No | [ ] ⏺  [ ] ⏺⏺  [ ] ⏺⏺⏺ |
| 29 | [ ] Headache  [ ] Migraine  [ ] Aura |  |  | _______________  _______________  _______________ | [ ] Y [ ] P [ ] No  [ ] Y [ ] P [ ] No  [ ] Y [ ] P [ ] No | [ ] ⏺  [ ] ⏺⏺  [ ] ⏺⏺⏺ |
| 30 | [ ] Headache  [ ] Migraine  [ ] Aura |  |  | _______________  _______________  _______________ | [ ] Y [ ] P [ ] No  [ ] Y [ ] P [ ] No  [ ] Y [ ] P [ ] No | [ ] ⏺  [ ] ⏺⏺  [ ] ⏺⏺⏺ |
| 31 | [ ] Headache  [ ] Migraine  [ ] Aura |  |  | _______________  _______________  _______________ | [ ] Y [ ] P [ ] No  [ ] Y [ ] P [ ] No  [ ] Y [ ] P [ ] No | [ ] ⏺  [ ] ⏺⏺  [ ] ⏺⏺⏺ |
| 32 | [ ] Headache  [ ] Migraine  [ ] Aura |  |  | _______________  _______________  _______________ | [ ] Y [ ] P [ ] No  [ ] Y [ ] P [ ] No  [ ] Y [ ] P [ ] No | [ ] ⏺  [ ] ⏺⏺  [ ] ⏺⏺⏺ |

| Day | Attack features | Intensity  1 to 10 | Lasting hours | Analgesics trade name (if took) | Effectiveness | Disability |
| --- | --- | --- | --- | --- | --- | --- |
| 33 | [ ] Headache  [ ] Migraine  [ ] Aura |  |  | _______________  _______________  _______________ | [ ] Y [ ] P [ ] No  [ ] Y [ ] P [ ] No  [ ] Y [ ] P [ ] No | [ ] ⏺  [ ] ⏺⏺  [ ] ⏺⏺⏺ |
| 34 | [ ] Headache  [ ] Migraine  [ ] Aura |  |  | _______________  _______________  _______________ | [ ] Y [ ] P [ ] No  [ ] Y [ ] P [ ] No  [ ] Y [ ] P [ ] No | [ ] ⏺  [ ] ⏺⏺  [ ] ⏺⏺⏺ |
| 35 | [ ] Headache  [ ] Migraine  [ ] Aura |  |  | _______________  _______________  _______________ | [ ] Y [ ] P [ ] No  [ ] Y [ ] P [ ] No  [ ] Y [ ] P [ ] No | [ ] ⏺  [ ] ⏺⏺  [ ] ⏺⏺⏺ |
